# Meta-research evaluating redundancy and use of systematic reviews when planning new studies in health research: a scoping review

**DOI:** 10.1186/s13643-022-02096-y

**Published:** 2022-11-15

**Authors:** Hans Lund, Karen A. Robinson, Ane Gjerland, Hanna Nykvist, Thea Marie Drachen, Robin Christensen, Carsten Bogh Juhl, Gro Jamtvedt, Monica Nortvedt, Merete Bjerrum, Matt Westmore, Jennifer Yost, Klara Brunnhuber, on behalf of the Evidence-Based Research Network

**Affiliations:** 1 Section Evidence-Based Practice, Department for Health and Function, Western Norway University of Applied Sciences, Inndalsveien 28, P.O.Box 7030, N-5020 Bergen, Norway; 2 Division of General Internal Medicine, Department of Medicine, Johns Hopkins University, Baltimore, MD USA; 3 Research and Analysis Department, University Library of Southern Denmark, Odense, Denmark; 4 Section for Biostatistics and Evidence-Based Research, the Parker Institute, Bispebjerg and Frederiksberg Hospital, Copenhagen, Denmark; 5 Research Unit of Rheumatology, Department of Clinical Research, University of Southern Denmark, Odense University Hospital, Odense, Denmark; 6 Department of Sports Science and Clinical Biomechanics, University of Southern Denmark, Odense, Denmark; 7 Department of Physiotherapy and Occupational Therapy, Herlev and Gentofte Hospital, Herlev, Denmark; 8 Faculty of Health Sciences, OsloMet, Oslo, Norway; 9 Faculty of Health and Social Science, Western Norway University of Applied Sciences, Bergen, Norway; 10 Research Unit of Nursing and healthcare, Institute of Public Health, Health, Aarhus University, Aarhus, Denmark; 11 The Centre of Clinical Guidelines, Department of Clinical Medicine, Aalborg University, Aalborg, Denmark; 12 The Danish Centre of Systematic Reviews - A JBI Centre of Excellence, The University of Adelaide, Adelaide, Denmark; 13 Health Research Authority, NHS, London, UK; 14 M. Louise Fitzpatrick College of Nursing, Villanova University, Villanova, PA USA; 15 Clinical Solutions, Elsevier Ltd., 125 London Wall, London, EC2Y 5AS UK

**Keywords:** Evidence-based research, Scoping review, Meta-research, Research on research, Systematicity, Transparency

## Abstract

**Background:**

Several studies have documented the production of wasteful research, defined as research of no scientific importance and/or not meeting societal needs. We argue that this redundancy in research may to a large degree be due to the lack of a systematic evaluation of the best available evidence and/or of studies assessing societal needs.

**Objectives:**

The aim of this scoping review is to (A) identify meta-research studies evaluating if redundancy is present within biomedical research, and if so, assessing the prevalence of such redundancy, and (B) to identify meta-research studies evaluating if researchers had been trying to minimise or avoid redundancy.

**Eligibility criteria:**

Meta-research studies (empirical studies) were eligible if they evaluated whether redundancy was present and to what degree; whether health researchers referred to all earlier similar studies when justifying and designing a new study and/or when placing new results in the context of earlier similar trials; and whether health researchers systematically and transparently considered end users’ perspectives when justifying and designing a new study.

**Sources of evidence:**

The initial overall search was conducted in MEDLINE, Embase via Ovid, CINAHL, Web of Science, Social Sciences Citation Index, Arts & Humanities Citation Index, and the Cochrane Methodology Register from inception to June 2015. A 2nd search included MEDLINE and Embase via Ovid and covered January 2015 to 26 May 2021. No publication date or language restrictions were applied.

**Charting methods:**

Charting methods included description of the included studies, bibliometric mapping, and presentation of possible research gaps in the identified meta-research.

**Results:**

We identified 69 meta-research studies. Thirty-four (49%) of these evaluated the prevalence of redundancy and 42 (61%) studies evaluated the prevalence of a systematic and transparent use of earlier similar studies when justifying and designing new studies, and/or when placing new results in context, with seven (10%) studies addressing both aspects. Only one (1%) study assessed if the perspectives of end users had been used to inform the justification and design of a new study. Among the included meta-research studies evaluating whether redundancy was present, only two of nine health domains (medical areas) and only two of 10 research topics (different methodological types) were represented. Similarly, among the included meta-research studies evaluating whether researchers had been trying to minimise or avoid redundancy, only one of nine health domains and only one of 10 research topics were represented.

**Conclusions that relate to the review questions and objectives:**

Even with 69 included meta-research studies, there was a lack of information for most health domains and research topics. However, as most included studies were evaluating across different domains, there is a clear indication of a high prevalence of redundancy and a low prevalence of trying to minimise or avoid redundancy. In addition, only one meta-research study evaluated whether the perspectives of end users were used to inform the justification and design of a new study.

**Systematic review registration:**

Protocol registered at Open Science Framework: https://osf.io/3rdua/ (15 June 2021).

**Supplementary Information:**

The online version contains supplementary material available at 10.1186/s13643-022-02096-y.

## Introduction

Science is cumulative; every new study should be planned, performed, and interpreted in the context of earlier studies ([1]; evbres.eu). At least this is how the ideal of science has been described [2–4]. Whether this ideal was being realised in science was publicly questioned as early as 1884 when Lord Rayleigh stated that “The work which deserves, but I am afraid does not always receive, the most credit is that in which discovery and explanation go hand in hand, in which not only are new facts presented, but their relation to old ones is pointed out” [5]. The lack of consideration of earlier studies when conducting new studies was analysed in a cumulative meta-analysis in 1992 by Lau et al. [6] who “found that a consistent, statistically significant reduction in total mortality ... was achieved in 1973, after only eight trials involving 2432 patients had been completed. The results of the 25 subsequent trials, which enrolled an additional 34,542 patients through 1988, had little or no effect on the odds ratio establishing efficacy, but simply narrowed the 95 percent confidence interval”. In the following years, several studies were published indicating that redundant and unnecessary studies have been conducted within different clinical areas such as cardiac diseases [7, 8], low back pain [9], dermatology [10], lung cancer [11], and dentistry [12].

In 2009, Robinson defended her doctoral thesis that showed that authors very rarely consider all earlier studies, but instead referring to only a small fraction of them or none at all [13, 14]. As Robinson wrote: “To limit bias, all relevant studies must be identified and considered in a synthesis of existing evidence. While the use of research synthesis to make evidence-informed decisions is now expected in health care, there is also a need for clinical trials to be conducted in a way that is evidence-based. *Evidence-based research* [emphasised here] is one way to reduce waste in the production and reporting of trials, through the initiation of trials that are needed to address outstanding questions and through the design of new trials in a way that maximises the information gained” [13]. Shortly after, an international network was established to promote an “Evidence-Based Research” (EBR) approach, that is, “the use of prior research in a systematic and transparent way to inform a new study so that it is answering questions that matter in a valid, efficient and accessible manner” (See: evbres.eu).

In a landmark series published in The Lancet in 2014, a group of researchers presented an overview of possible reasons for waste or inefficiency in biomedical research [15]. They described five overall areas of concern: (1) research decisions not based on questions relevant to users of research; (2) inappropriate research design, methods, and analysis; (3) inefficient research regulation and management; (4) not fully accessible research information; and (5) biased and not usable research reports. Several of these reasons for waste or inefficient research can be related to a lack of evidence-based research, with researchers addressing low-priority research, not assessing important outcomes, rarely using systematic reviews (SRs) to inform the design of a new study, and new results not being interpreted in the context of the existing evidence base. In this paper, we refer to such failings as questionable research practices (QRPs) [16]. A QRP does not constitute “research misconduct”, i.e. fabrication or falsification of data and plagiarism, but a failure to align with the principles of scientific integrity.

Even though numerous factors can influence whether researchers perform and publish unnecessary research, in this scoping review we have chosen to focus on meta-research studies evaluating the frequency and characteristics of the following QRPs: (A) authors publishing redundant studies; (B) authors not using the results of a systematic and transparent collection of earlier similar studies when justifying a new study; (C) authors not using the results of a systematic and transparent collection of earlier similar studies when designing a new study; (D) authors not systematically and transparently placing new results in the context of existing evidence; and (E) authors not systematically and transparently using end user’s perspectives to inform the justification of new studies, the design of new studies, or the interpretation of new results.

Our search identified no previous scoping review of meta-research studies evaluating redundancy in biomedical research. As the first scoping review of its kind, our aim was to (A) identify meta-research studies evaluating if redundancy was present, and the prevalence of redundancy within biomedical research, and (B) to identify meta-research studies evaluating if researchers had been trying to minimise or avoid redundancy. It was further our intention to examine the extent, variety, and characteristics of included meta-research studies to identify any research gaps that could be covered by future meta-research.

## Methods

### Protocol and registration

A protocol was registered at Open Science Framework: https://osf.io/3rdua/ (15 June 2021). The reporting of this scoping review follows the PRISMA extension for scoping reviews [17] (see also Additional File 4_PRISMA Checklist for Scoping Reviews filled in).

### Eligibility criteria

We included meta-research studies evaluating the presence and characteristics of the following QRPs: (A) authors publishing redundant studies; (B) authors not using the results of a systematic and transparent collection of earlier similar studies when justifying a new study; (C) authors not using the results of a systematic and transparent collection of earlier similar studies when designing a new study; (D) authors not systematically and transparently placing new results in the context of existing evidence; and (E) authors not systematically and transparently using end user’s perspectives to inform the justification of new studies, the design of new studies, or the interpretation of new results.

We did not define redundancy ourselves but noted the definitions the study authors were using.

### Information sources and search

The initial overall search was conducted in MEDLINE via both PubMed and Ovid, Embase via Ovid, CINAHL via EBSCO, Web of Science (Science Citation Index Expanded (SCI-EXPANDED), Social Sciences Citation Index (SSCI), Arts & Humanities Citation Index (A&HCI), and Cochrane Methodology Register (CMR, Methods Studies) from inception to June 2015. No restrictions on publication date and language were applied.

As there are no standard search terms for meta-research studies (many such studies never used the word “meta-research” or its synonyms) nor for studies evaluating the use of an evidence-based research approach, the first search results were sensitive but lacked precision. A second, more focused search was performed in May 2021 in MEDLINE and Embase via Ovid covering the period from January 2015 to 26 May 2021. To evaluate the recall of the 2nd search strategy, we ran this new search strategy also for the timeframe up to 2015 and found that it would have picked up all studies we had included from our first search. For a more detailed description of the 1st search, see Appendix 1, and Appendix 2 for the 2nd search.

In addition, we checked reference lists of all included studies and asked experts within the field for any missed relevant literature.

### Selection of sources of evidence

The search results were independently screened by two people who resolved disagreements on study selection by consensus and as needed, by discussion with a third reviewer. Full-text screening was performed by four reviewers independently who resolved disagreements on study selection by consensus and discussion.

### Data charting process

We developed and pilot tested a data-charting form. Two reviewers independently extracted data, discussed the data, and continuously updated the data charting form in an iterative process.

### Data items

All studies were categorised according to the following framework that distinguishes between meta-research studies evaluating the presence of the problem, redundant and unnecessary studies, and studies evaluating whether an evidence-based research (EBR) approach had been used (see Table 1).

**Table 1:** Framework to categorise included studies

**The problem:** Redundancy	**Data material**	**Overall design**	**Methods**	**Metrics**
**Possible solutions:** Avoiding or minimising the questionable research practices listed in the section “Eligibility criteria”	**Data material**	**Overall design**	**Methods**	**Metrics**

The data-charting form also included information related to the publication (i.e. authors, publication year, country of 1st author, journal, and publishing house), the topic (health domain), data material, overall design, and methods (e.g. sources of data, outcomes), results, and conclusions.

The author names were used to create a bibliometric map in the software VOSViewer (Leiden University’s Centre for Science and Technology Studies (CWTS), VOSviewer version 1.6.17). The map visualised the diversity of published studies, i.e. whether the meta-research identified was published by a small group of authors. The map displays one node/circle for each of the authors included in this review. Larger nodes indicate more relevant articles published by that particular author, and each node is linked with the nodes of its co-authors. On the large map, this linking is illustrated by the nodes being placed close together, so that they form an “island”. Thicker lines between authors mean more papers written together.

### Research gaps in meta-research

To identify research gaps within meta-research related to evidence-based research, we (A) counted the number of studies evaluating each of the QRPs listed in the section “Eligibility criteria”; (B) combined a list of research topics with the three main aspects of evidence-based research: justification (Justification) and design (Design) of a new study and the interpretation of the new results in context (Context), as well as measurement of redundancy; (C) combined a list of data materials being used in the meta-research studies with the above-mentioned three aspects of evidence-based research, as well as redundancy; and (D) identified the type of health domains covered by redundancy and the three aspects of evidence-based research.

## Results

### Selection of evidence sources

**Fig. 1:**
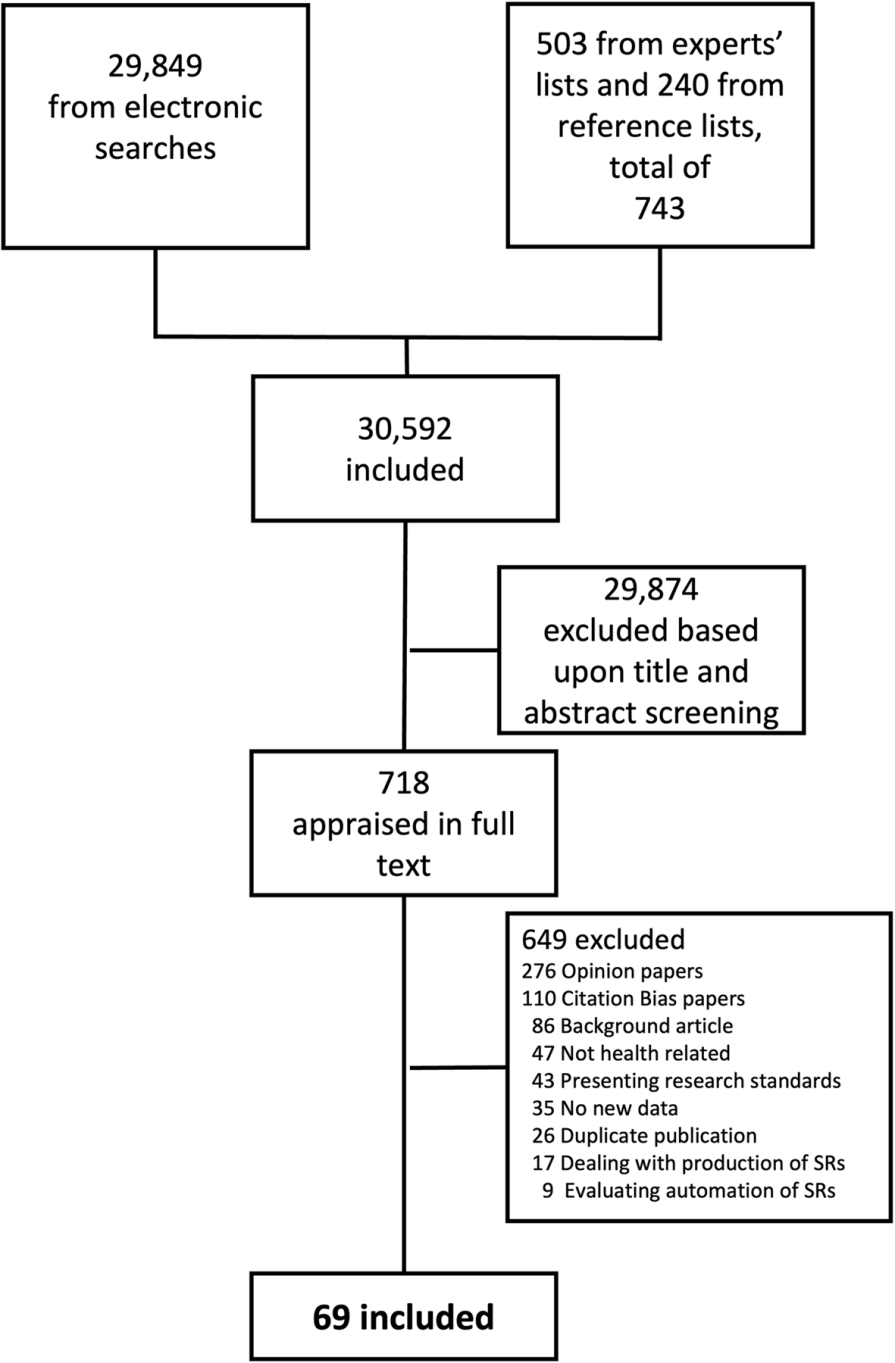
PRISMA flowchart

We identified a total of 30,592 unique citations and included 69 original meta-research studies in our analysis (see Fig. 1; Additional Material 11).

### Characteristics of evidence sources

The specific characteristics of the included studies are presented in Table 2. The studies were published in the period from 1981 to 2021, with a peak of 10 studies published in 2017 (Supplementary Material 1). Only one study was published in the 1980s, while the majority (60 studies; 87%) were published after 2000.

**Table 2:** Characteristics of included studies

Author and year	Country^1^	Health Field^2^	Data material	Measures
Antman 1992 [7]	USA	Circulation and breathing	Primary studies	Redundancy
Ban 2017 [8]	UK	Circulation and breathing	Primary studies and researchers	Use of the EBR approach (JUST, SIM)^a^
Baum 1981	USA	Abdomen and endocrine	Primary studies	Redundancy
Bauman 2017	Australia	Public health and health systems	Primary studies	Redundancy
Bhurke 2015	UK	No specific medical specialty	Proposal funding	Use of the EBR approach (JUST, DESIGN)^a^
Blanco-Silvente 2019	Spain	Mental health and neuroscience	Primary studies	Redundancy
Bolland 2018	New Zealand	Musculoskeletal, oral, skin, and sensory	Primary studies	Redundancy
Bolland 2018 (part 2)	New Zealand	Musculoskeletal, oral, skin, and sensory	Primary studies	Use of the EBR approach (JUST, DESIGN)^a^
Brockington 2017	UK	Mental health and neuroscience	Primary studies	Use of the EBR approach (JUST, SIM)^a^
Chalmers 1996	USA	Children and families	Primary studies	Redundancy
Chapman 2019	UK	No specific medical specialty	Primary studies	Use of the EBR approach (DESIGN)^a^
Chiu 2021	USA	Cancer	Primary studies	Redundancy
Chow 2017	Canada	No specific medical specialty	Primary studies	Use of the EBR approach (JUST)^a^
Chow 2019	Canada	Cancer	Primary studies	Redundancy
Chow 2020	Canada	Cancer	Primary studies	Redundancy
Clarke M 2002	UK	No specific medical specialty	Primary studies	Use of the EBR approach (CONTXT)^a^
Clarke M 2014	UK	No specific medical specialty	Systematic reviews	Redundancy
Clarke M 1998 [3]	UK	No specific medical specialty	Primary studies	Use of the EBR approach (CONTXT)^a^
Clarke M 2007	UK	No specific medical specialty	Primary studies	Use of the EBR approach (JUST, DESIGN, CONTXT)^a^
Clarke M 2010	UK	No specific medical specialty	Primary studies	Use of the EBR approach (DESIGN, CONTXT)^a^
Clarke M 2013	UK	No specific medical specialty	Primary studies	Use of the EBR approach (JUST, DESIGN, CONTXT)^a^
Clayton 2017	UK	No specific medical specialty	Researchers (Conference delegates)	Use of the EBR approach (JUST, DESIGN, CONTXT)^a^
Conde-Taboada 2014 [10]	Spain	Musculoskeletal, oral, skin, and sensory	Primary studies	Use of the EBR approach (JUST)^a^
Coomarasamy 2006	UK	Children and families	Primary studies	Redundancy
Cooper 2005	UK	No specific medical specialty	Researchers	Use of the EBR approach (DESIGN)^a^
De Meulemeester 2018	Canada	No specific medical specialty	Primary studies	Use of the EBR approach (JUST)^a^
Engelking 2018	Croatia	Acute and emergency care	Primary studies	Use of the EBR approach (JUST)^a^
Fergusson 2005	Canada	Circulation and breathing	Primary studies	Redundancy Use of the EBR approach (JUST, SIM)^a^
Fergusson 2018 [18]	Canada	No specific medical specialty	Primary studies	Use of the EBR approach (END-USER)^a^
Goudie 2010	UK	No specific medical specialty	Primary studies	Use of the EBR approach (JUST, DESIGN, CONTXT)^a^
Habre 2014 [19]	Switzerland	Musculoskeletal, oral, skin, and sensory	Primary studies	Redundancy (due to design error) Use of the EBR approach (DESIGN)^a^
Helfer 2015	Germany	No specific medical specialty	Systematic reviews	Use of the EBR approach (JUST, SIM, CONTXT)^a^
Henderson 1995	USA	Circulation and breathing	Primary studies	Redundancy
Hoderlein 2017 [20]	Germany	Various sciences including healthcare	Primary studies	Use of the EBR approach (JUST, CONTXT)^a^
Ivers 2014	Canada	Public health and health systems	Primary studies	Redundancy
Jia 2021 [21]	USA	Circulation and breathing	Primary studies	Redundancy
Johnson 2020	USA	Acute and emergency care	Primary studies	Use of the EBR approach (JUST)^a^
Jones 2013	UK	No specific medical specialty	Proposal funding	Use of the EBR approach (JUST, DESIGN)^a^
Joseph 2018.	Australia	No specific medical specialty	Protocol RECs	Use of the EBR approach (JUST)^a^
Jüni 2004	Switzerland	Circulation and breathing	Primary studies	Redundancy
Ker 2012	UK	Circulation and breathing	Primary studies	Redundancy
Ker 2015 [22]	UK	Musculoskeletal, oral, skin, and sensory	Primary studies	Redundancy Use of the EBR approach (JUST)^a^
Lau 1992 [6]	USA	Circulation and breathing	Primary studies	Redundancy
Love 2018	UK	Public health and health systems	Primary studies	Redundancy
Murad 2017	Switzerland	No specific medical specialty	Primary studies	Redundancy
Paludan-Muller 2019	Denmark	No specific medical specialty	Protocol RECs	Use of the EBR approach (JUST, DESIGN)^a^
Pandis 2016 [12]	Switzerland	No specific medical specialty	Published protocols	Use of the EBR approach (JUST, DESIGN)^a^
Park 2017	Korea	Cancer	Systematic reviews	Redundancy (overlapping meta-analyses) Use of the EBR approach (JUST, SIM)^a^
Poolman 2007	The Netherlands	Acute and emergency care	Primary studies	Redundancy
Rauh 2020	USA	Children and families	Primary studies	Use of the EBR approach (JUST)^a^
Riaz 2016	USA	No specific medical specialty	Systematic reviews	Redundancy (overlapping meta-analyses)
Robinson 2014	USA	No specific medical specialty	Primary studies	Use of the EBR approach (JUST, SIM)^a^
Robinson 2011 [14]	USA	No specific medical specialty	Primary studies	Use of the EBR approach (JUST, SIM)^a^
Rosenthal 2017	Switzerland	No specific medical specialty	Primary studies	Use of the EBR approach (JUST, DESIGN, CONTXT)
Ross 2009	USA	Circulation and breathing	Primary studies	Redundancy
Sawin 2016	USA	Circulation and breathing	Primary studies	Use of the EBR approach (JUST, SIM)^a^
Seehra 2021	UK	Musculoskeletal, oral, skin, and sensory	Primary studies	Use of the EBR approach (JUST)^a^
Shepard 2021	USA	Abdomen and endocrine	Primary studies	Use of the EBR approach (JUST)^a^
Sheth 2011	Canada	Acute and emergency care	Primary studies	Use of the EBR approach (JUST, SIM)^a^
Sigurdson 2020	USA	Public health and health systems	Systematic reviews	Redundancy (overlapping meta-analyses) Use of the EBR approach (JUST, SIM)^a^
Sinclair 1995	Canada	Children and families	Primary studies	Redundancy
Siontis 2013	USA	No specific medical specialty	Systematic reviews	Redundancy (overlapping meta-analyses) Use of the EBR approach (JUST)^a^
Smith 1997	UK	Circulation and breathing	Primary studies	Use of the EBR approach (DESIGN)^a^
Storz-Pfennig 2017	Germany	No specific medical specialty	Systematic reviews	Redundancy
Torgerson 2020	USA	Musculoskeletal, oral, skin, and sensory	Primary studies	Use of the EBR approach (JUST)^a^
Vergara-Merino 2021	Chile	No specific medical specialty	Primary studies	Redundancy
Verhagen 2019	The Netherlands	Musculoskeletal, oral, skin, and sensory	Primary studies	Redundancy
Walters 2020	USA	No specific medical specialty	Primary studies	Use of the EBR approach (JUST)^a^
Whiting 1995	USA	Cancer	Primary studies	Redundancy

All references are listed in Additional file 1, additional material 11

^a^Questionable research practices evaluated:

JUST, authors do not use the results of a systematic and transparent collection of earlier similar studies when justifying a new study; SIM, authors of a scientific study do not refer to all earlier similar studies; DESIGN, authors do not use the results of a systematic and transparent collection of earlier similar studies when designing a new study; END-USER, authors do not use the results of a systematic and transparent collection of the new research projects’ end user’s perspectives to inform the justification and design of the new study; CONTXT, authors do not systematically and transparently place new results in the context of existing evidence

The first authors of the included studies were based at institutions in 13 different countries. Fifty-eight percent of identified studies were published in the UK and USA (20 from each) (see Supplementary Material 2).

The included studies evaluated various health domains (Table 5), with a large proportion of studies (28 studies, 40.6% of all) cutting across all medical specialties.

**Fig. 2:**
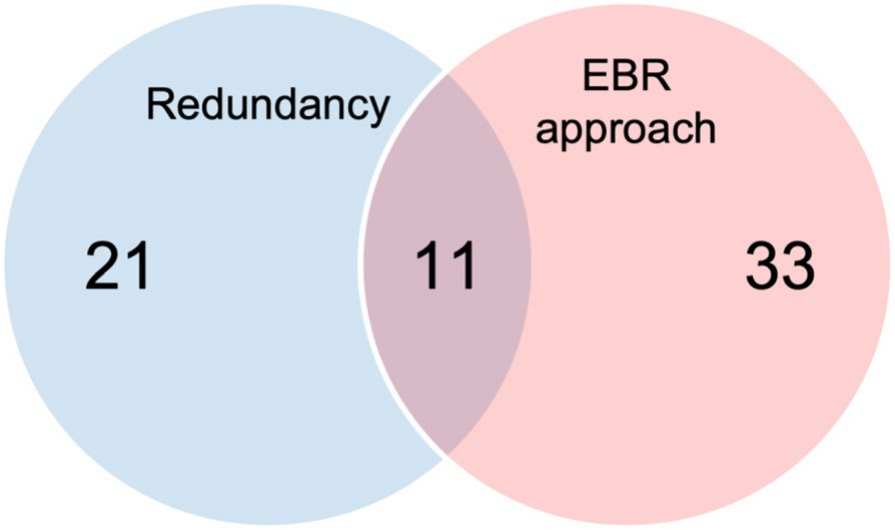
Venn diagram indicating the number of studies that evaluated either redundancy, use of the EBR approach, or both

A Venn diagram was used to indicate the number of studies investigating if authors were publishing redundant studies, if authors were using an EBR approach to avoid QRPs and any combination hereof (Fig. 2).

The information about data material, overall designs, used methods, and metrics, as planned (see Table 1), was identified and is listed in Table 3.

**Table 3:** The framework to categorise included studies filled in based upon included studies

	Data material	Overall design	Methods	Metrics
**The problem:** Redundancy	1. Primary studies 2. Systematic reviews 3. Funding proposals 4. REC proposals 5. Published protocols 6. Researchers	1. Systematic review 2. Cross-sectional study 3. Other observational study 4. Qualitative study	1. Citation analysis 2. Content analysis 3. Cut-off analysis 4. Survey	1. The number of overlapping meta-analyses 2. Impact of SR on design of subsequent research 3. Description of cumulative meta-analyses
**Possible reasons for problem:** Presence of QRPs	1. Primary studies 2. Systematic reviews 3. Funding proposals 4. REC proposals 5. Published protocols 6. Researchers	1. Systematic review 2. Cross-sectional study 3. Other observational study 4. Qualitative study 5. Randomised study	1. Citation analysis 2. Content analysis 3. Survey	1. Number of studies citing SRs 2. Number of studies citing available/relevant SRs 3. Number of similar original studies cited 4. Number of studies citing similar studies 5. Number of studies citing prior SR 6. Number of studies citing SRs or original studies 7. Number of available / relevant SRs that were cited 8. Number and type of studies cited

*REC* Research Ethic Committee

In studies evaluating redundancy, three metrics were used for content analyses (see Table 3). The number of overlapping meta-analyses refers to meta-research studies identifying systematic reviews that cover the same topic and hence overlap with each other. One of the included meta-research studies stated that “Systematic reviews often provide a research agenda to guide future research” [19]; thus, meta-research studies could use this as a metric, i.e. evaluate if authors made any changes to the trial design after publication of the research agenda. The majority of the studies evaluating redundancy used cumulative meta-analyses in one way or another; thus, the overall metric would be a description of cumulative meta-analyses indicating redundancy (see also Supplementary Material 3).

A crucial element in using a cumulative meta-analysis is the selection of cut-offs, i.e. the factor determining if a study would be considered to be redundant. A cut-off could also be used without performing a cumulative meta-analysis, but instead stating that “redundant clinical trials were defined as randomized clinical trials that initiated or continued recruiting after 2008” where a clinical guideline was published [21]. Different criteria were used for cut-off analyses, with four of them using cumulative meta-analysis (*p-*value, visual inspection of a forest plot, trial sequential analysis, and failsafe ratio), and the others either extended funnel plot, number of similar trials published after a trial is stopped early for benefit, number of studies published after established “high” certainty of evidence, or the number of studies published after established guidelines had established certainty of evidence (see also Supplementary Material 3).

The number of studies using different cut-off criteria was too low to identify potential differences by cut-off type. For an overview of the data materials, study designs, and study methods used in studies evaluating redundancy and the use of an EBR approach to minimise or avoid redundancy, see Supplementary Materials 7–9.

In studies evaluating the use of an evidence-based research approach to minimise or avoid redundancy, eight different metrics were used to perform a citation analysis (see Table 3 and Supplementary Material 4). Furthermore, ten metrics were used to perform a content analysis (see Table 3 and Supplementary Material 4).

Only one study performed a survey asking researchers about the use of SRs when justifying and designing new studies (see Supplementary Material 4).

**Fig. 3:**
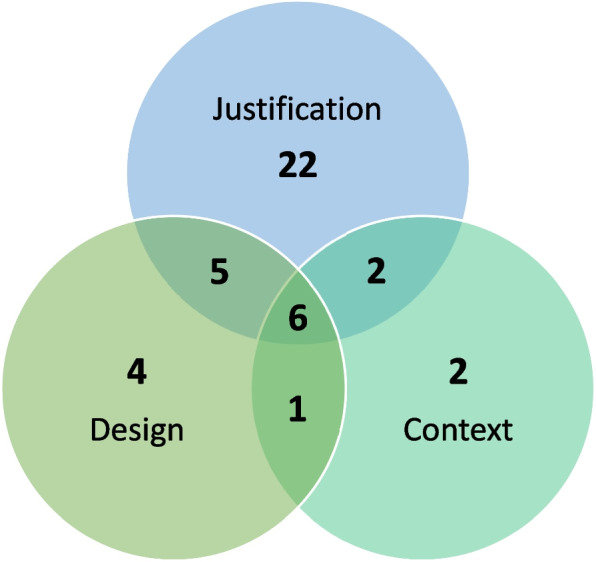
Venn diagram indicating the number of studies that investigated the following questionable research practices; “Justification”—authors do not use the results of a systematic and transparent collection of earlier similar studies when justifying a new study; “Design”—authors do not use the results of a systematic and transparent collection of earlier similar studies when designing a new study; and “Context”—authors do not systematically and transparently place new results in the context of existing evidence, and any combinations thereof. Ten of the studies investigating whether authors use the results of a systematic and transparent collection of earlier similar studies when justifying a new study also evaluated whether authors of a scientific study referred to all earlier similar studies. One of the six studies in the middle section also investigated whether authors used the results of a systematic and transparent collection of the new research projects’ end user’s perspectives to inform the justification and design of the new study

The studies evaluating the use of the EBR approach to minimise or avoid redundancy evaluated three different aspects; (A) authors do not use the results of a systematic and transparent collection of earlier similar studies when justifying a new study; (B) authors do not use the results of a systematic and transparent collection of earlier similar studies when designing a new study; and (C) authors do not systematically and transparently place new results in the context of existing evidence. Figure 3 indicates the number of studies evaluating each of these questionable research practices. As only one study evaluated whether authors used the results of a systematic and transparent collection of the new research projects’ end user’s perspectives to inform the justification and design of the new study, it was not included in Fig. 3.

### Bibliographic mapping

**Fig. 4:**
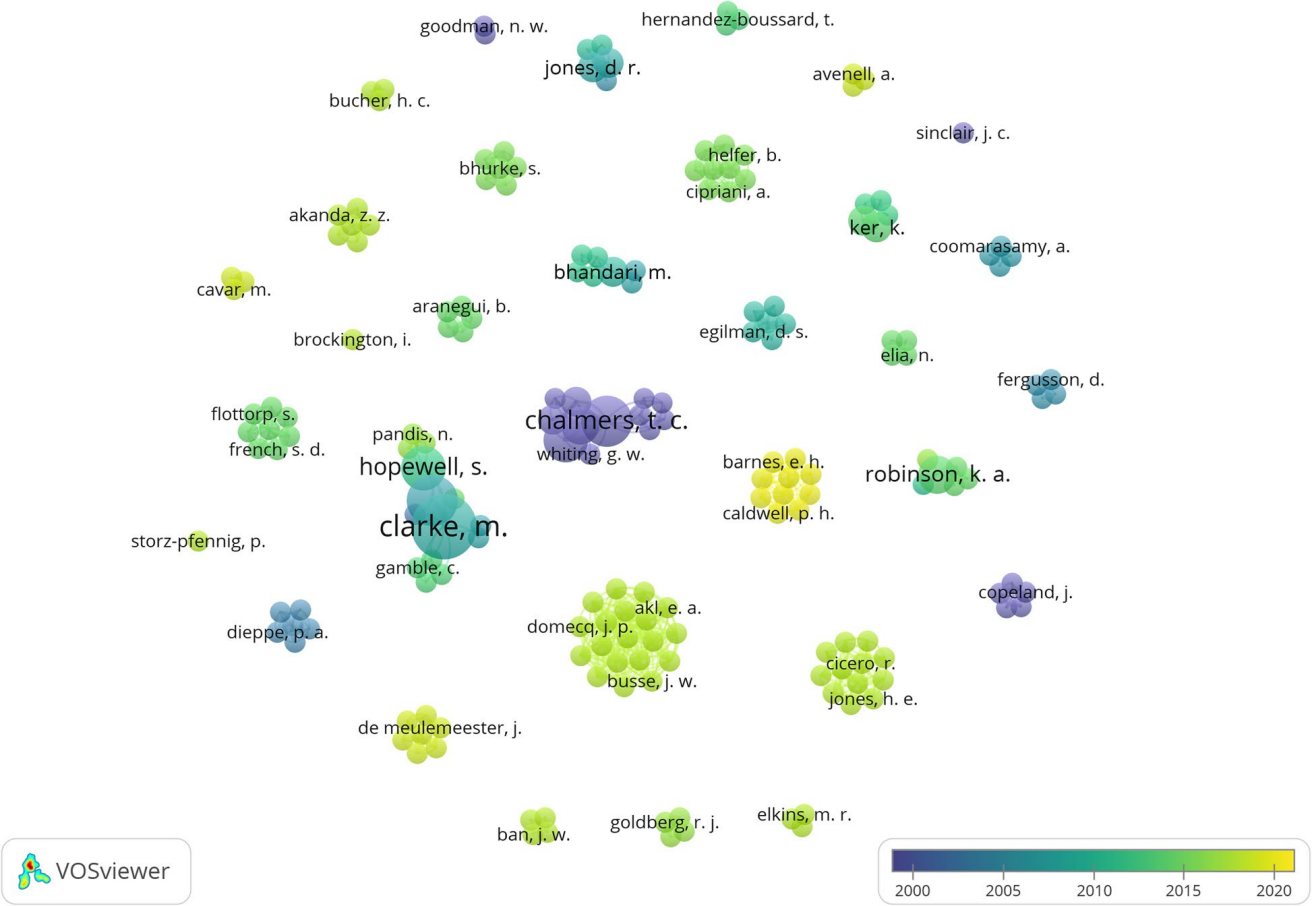
Bibliometric map

Bibliographic mapping revealed 41 independent author groups that had conducted the included meta-research studies (see Fig. 4). Because of the high number of co-author islands (*n* = 41), the total map is quite large. The two smaller maps (see Supplementary Material 5 and 6) have zoomed in on the two largest islands in terms of the number of published papers.

This bibliographic mapping of included studies indicates that only a small number of studies were published with the same authors involved. Most studies were conducted by groups of researchers—and some individual researchers—working in isolation from each other (see also Table 2). Still, we cannot exclude the possibility that some of these islands may be part of larger collegial groups and that some of the authors from different islands may have co-authored papers on topics not included in this scoping review. Based on the current findings, however, there is no indication that the identified studies were published by a small group of authors and/or research groups.

### Research gaps

Tables 4, 5, and 6 present the types of research gaps relating to the methods used in the included meta-research studies.

**Table 4:**
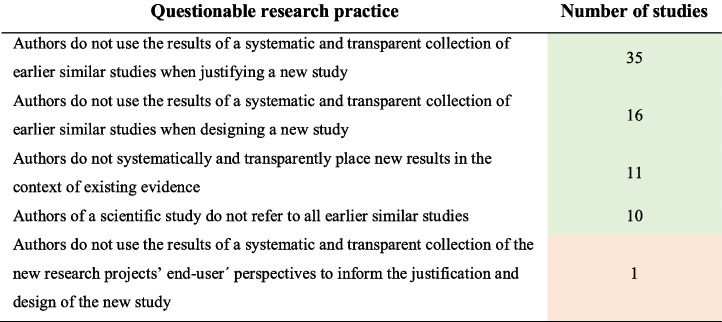
The number of studies evaluating questionable research practice

**Table 5:**
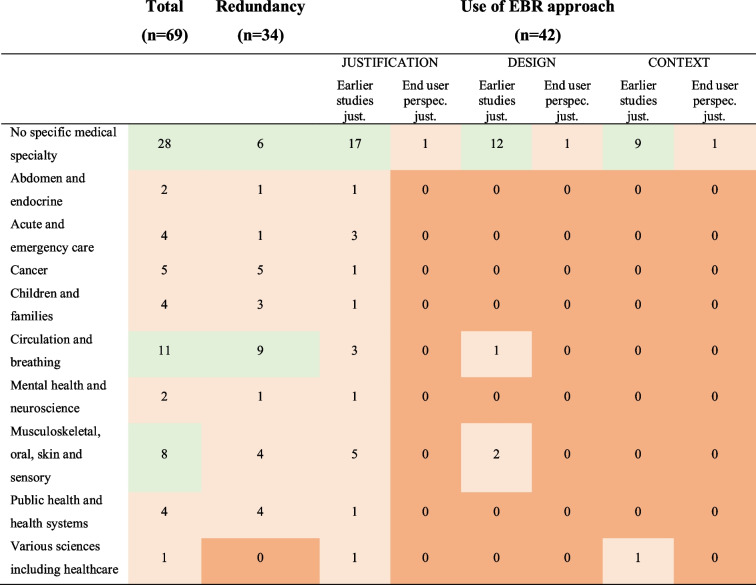
Types of health domains covered by the included studies. Health domains inspired by Cochrane’s eight Review Group Networks (https://www.cochrane.org/news/overview-cochranes-eight-review-group-networks)

**Table 6:**
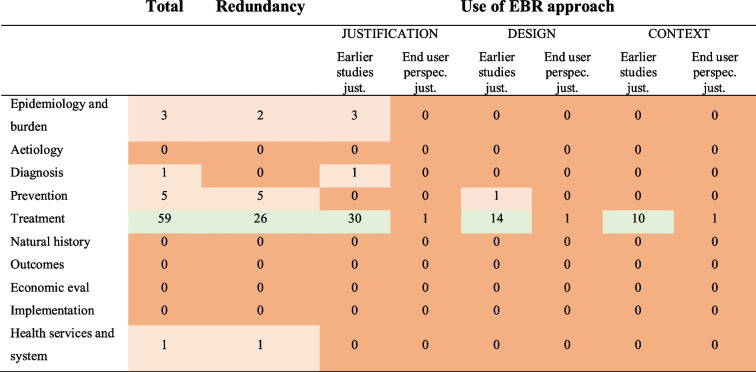
Combined list of research topics [23]

Table 4 presents the number of studies evaluating the four QRPs (see above). Only one study evaluated whether authors used the results of a systematic and transparent collection of end users’ perspectives to inform the justification and design of new studies. Tables 5 and 6 present lists of research topics and health domains combined with the three aspects of evidence-based research: justification (Justification) and design (Design) of a new study and the interpretation of the new results in context (Context), as well as measurement of redundancy. The list of research topics was inspired by Bourne et al. [23]. The list of health domains was based on Cochrane’s eight Review Group Networks (Cochrane.org). Additional tables (see Supplementary Material) combine lists of the data materials, study designs, and analysis methods used in the meta-research studies with the three aspects of evidence-based research, as well as redundancy.

Fields marked in the tables in light green indicate that the relevant issues had been evaluated by several studies (6 or more), with light red indicating only a few studies (5 or less), and fields marked in red indicate that no studies had been identified. The sum of studies listed in the tables is higher than the total number of included studies because several studies have evaluated more than one questionable research practice as part of the same study.

### Conclusions of included studies

We prepared a list of conclusions extracted from the included studies (See Supplementary Material 10). Twenty-three studies had concluded that redundancy was present among similar clinical studies, and three studies had identified redundancy among similar SRs. Fifteen studies reported no or poor use of SRs to inform justification of a new study, while six studies showed no or poor use of SRs to inform design, and seven studies demonstrated no or poor use of SRs when placing new results in the context of existing research.

## Discussion

We identified 34 meta-research studies that evaluated whether redundancy existed among similar studies and 42 studies that evaluated whether authors of clinical studies had used a systematic and transparent approach to avoid redundancy in health research, with seven studies addressing both aspects. There is a clear indication of high prevalence of redundancy and low prevalence that researchers had tried to minimise or avoid redundancy from 28 studies evaluating across different medical specialties.

Despite the 69 meta-research studies included in this scoping review, there is a dearth of information for most health domains and research topics. Only a single meta-research study evaluated whether end users were involved in the justification and design of a new study [18]. Almost all meta-research studies focused on research evaluating the effect of a treatment. Only six studies evaluated research dealing with questions on epidemiology or disease burden, five with disease prevention, and only one with diagnostic issues. This means that a large number of research topics have never been evaluated in relation to the possibility of redundancy in research or whether researchers have used a systematic and transparent approach when justifying and designing new studies and when placing new results in the context of existing evidence, including aetiology, natural history, outcomes, economic evaluations, implementation, and health services and system.

Most meta-research had analysed published papers of original studies (typically treatment evaluation studies), while very few focused on other sorts of research documents such as funding and ethic committee proposals or published protocols. Only four studies explored redundancy in the production of SRs, and none evaluated whether researchers had used a systematic and transparent approach when justifying and designing a new SR, or when placing new results in the context of the existing evidence. Finally, studies applied widely varying methods (cut-off points) and definitions to evaluate whether redundancy was present. A frequency statistic approach was used most often, whereas no study utilised a Bayesian approach.

The seriousness of the problem evaluated in the present scoping review is highlighted in the conclusions of the studies and can be summed up in the following results: Evidence shows that researchers make no or poor use of SRs when justifying and/or designing new studies, or when placing new study results in the context of existing similar research.

### Strengths and limitations of this scoping review

Both the long time it took to prepare this scoping review and the large number of hits in the literature search (>30.000 hits) indicate the immense challenges of identifying relevant studies. This is further supported by the high proportion of studies identified via direct contact with experts, reading of reference lists, and additional citation searches. The reasons are at least two-fold. First, it remains difficult to identify research-on-research studies, partly due to the lack of a standardised naming convention for these kind of studies (for example: meta-epidemiology, research on research, meta-research, metascience, and science of science) and the fact that many authors never define their studies as meta-research studies in the first place. Secondly, it is an even greater challenge to identify studies evaluating the specific topics related to the only recently defined evidence-based research concept (i.e. studies identifying redundancy or unnecessary studies, and studies evaluating whether authors are using a systematic and transparent approach when justifying and designing new studies, and when placing new results in the context of the existing evidence). As the presence of our two search strategies indicates, we had to undertake initial searching and screening before we could prepare a sufficiently precise search strategy (see appendix 1 and 2).

Thanks to our extensive literature searches, we are assured that this is indeed the first scoping review of meta-research evaluating redundancy in health research and different ways to minimise such redundancy.

We deemed the problem of citation bias as beyond the scope of this scoping review. However, identifying studies that evaluate the reasons why researchers select references in their publications could provide important answers as to the root causes of redundancy and why researchers rarely use a systematic and transparent approach when planning and interpreting new studies. Hence, a further scoping review is in preparation that focuses on studies evaluating citation bias and other biases related to the citing of other publications.

It is also beyond the aim of a scoping review to report the size of reported QRPs, but the fact that a large and very diverse sample of meta-research studies showed that an evidence-based research approach is rarely used indicates that a fundamental problem exists with the way new research is currently planned and interpreted. This is corroborated by the finding that all identified studies consistently reported the same lack of using a systematic and transparent approach when justifying and designing new studies or when placing new findings in the context of the existing body of knowledge, even though these studies had been prepared by a large and diverse group of authors covering many health domains.

As almost all identified studies consistently showed redundancy or poor use of the evidence-based research approach, publication bias cannot be ruled out. It is possible that studies with positive results, i.e. identifying redundancy or bad behaviour, were more likely to have been published. We identified only two studies that did not report a problem: Ker 2015 [22] and Hoderlein 2017 [20]. The first of these studies found no reason to assume that the QRP of authors not using the results of a systematic and transparent collection of earlier similar studies when justifying a new study was present as the authors argued that the low quality of earlier studies limited generalisability of results and hence justified yet another study [22]. It is of note, however, that the number of new studies actually increased after a SR was published, and that, as the authors pointed out, “over half of trials cited at least one of the existing SRs suggests that ignorance of the existing evidence does not fully explain ongoing trial activity” [22]. The authors also argued that new studies were justified as new patient groups had been added in the clinic to use the treatment. The possibility that authors citing the SR were not utilising it to justify the new study was not considered. To evaluate this aspect, the authors of the meta-research studies would not only need to read each Background or Discussion section but also interpret the sentences related to citing a SR. This would require not only careful text analysis but also interviews with the authors themselves to find out about their reasons for selecting cited references. Based on the results from earlier studies, these reasons can be manyfold, with only a few related to justifying or designing a new study [24, 25].

### Implications for research practice

This scoping review does not comprehensively evaluate all reasons for redundant research, but our results clearly indicate that researchers hardly ever use a systematic and transparent (“Evidence-Based Research”) approach when planning and interpreting new studies. Even though this explains to a large extent the publication of redundant studies, it is unclear why researchers, who had been trained to be systematic and transparent in everything they do while performing research, are not being similarly systematic and transparent during the planning and interpreting phases of the research process. One reason could be a lack of knowledge about the problem, calling for further education of researchers. Such educational programmes (already taking place in EVBRES (evbres.eu)) should include modules that increase learners’ understanding of the need for systematic reviews and how to use them to inform the justification and design of studies, and when interpreting new results in the context of existing evidence.

### Implications for future meta-research

Our analyses showed that only one meta-research study evaluated the inclusion of end users’ perspectives when justifying or designing a new study. Many more studies are needed to evaluate end user involvement in these fundamental research aspects and how end users’ perspectives can be best obtained. Furthermore, only a few studies evaluated redundancy in published SRs, even though some studies have indicated a large increase in the production of SRs over time [26]. In addition, we have identified at least nine different ways of defining when no further studies are needed (see Supplementary Material 4). Most of these definitions have used a frequency statistic approach to determine dichotomy cut-off points. However, as stated in a report from the Cochrane Scientific Committee, SR authors should be discouraged from drawing binary interpretations of effect estimates [27]. Even with the grading of evidence as a method to avoid this binary approach, there is a need for more precise and reliable methods [28, 29].

Additionally, meta-research studies are needed to evaluate how new studies should be justified when applying for ethical approval or funding, or when preparing a study protocol. This would make it easier to evaluate the importance of using a systematic and transparent approach during ethical or funding approval in the interest of both research ethic committees and funding agencies.

Finally, as publication bias could not be ruled out, larger meta-research studies cutting across different health domains are needed to evaluate whether publication bias really exists.

Most of the included meta-research studies analysed the content of published original papers. Even though this can provide a good overview of the situation, the data extracted from original papers rarely explains why researchers have not used an evidence-based research approach. Surveys and qualitative studies are needed to understand the underlying incentives or motivational factors, and the facilitators and barriers behind the lack of a systematic and transparent approach during the justification and design phases of planning a new study.

“These initiatives have mainly emerged from the biomedical sciences and psychology, and there is now an increasing need for initiatives tailored to other research disciplines and cultures.” [30]. This scoping review has focused solely upon research within health. Considering the characteristics of the described problem (too much redundancy and too little systematicity and transparency while planning new studies and interpreting new results), this problem could exist to a similar level in other research disciplines and faculties, necessitating relevant research within social science, natural science, and the humanities.

## Supplementary Information

**Additional file 1: Additional Material 1.** Figure showing number of studies published per year. **Additional Material 2.** Figure indicates the number of studies from each country, measured as the country affiliation of 1st authors. **Additional Material 3.** Metrics used in studies evaluating redundancy. N = Number of studies. The total number is higher than the actual number of studies evaluating redundancy, because most studies have used more than one metric. **Additional Material 4.** Table presenting the metrics used in studies evaluating the use of the EBR approach to minimise or avoid redundancy. N = Number of studies. The total number is higher than the actual number of studies evaluating the use of the EBR approach because most studies have used more than one metric. **Additional Material 5.** Bibliographic map, the M. Clarke group. **Additional Material 6.** Bibliographic map, the T.C. Chalmers group. **Additional Material 7.** Table listing the data materials used in the included studies. (Note that “Primary studies” include papers using various kinds of studies as data material, including some systematic reviews.) Fields marked in light green indicate several studies (6 or more), those in light red indicate few studies (5 or less), and those marked in red indicate no studies. The sum of studies evaluating redundancy/use of EBR approach is higher than the total number, because several studies have evaluated more than one research question. Also, one included paper evaluating the use of the EBR approach [8] used both primary studies and researchers as data material and was therefore counted twice in the table. **Additional Material 8.** Table listing study designs used in the included studies. Fields marked in light green indicate several studies (6 or more), those in light red indicate few studies (5 or less), and those marked in red indicate no studies. The sum of studies evaluating redundancy/use of EBR is higher than the total number, because several studies have evaluated more than one research question. Also, one included paper evaluating the use of the EBR approach [8] used both cross-sectional and another observational study designs and was therefore counted twice in the table. **Additional Material 9.** Table listing analysis methods used in the included studies. Fields marked in light green indicate several studies (6 or more), those in light red indicate few studies (5 or less), and those marked in red indicate no studies. Note that many of the included studies used more than one analysis method and investigated more than one research question. For that reason, studies are counted several times in the table, and their sum is much higher than the total number of included papers. **Additional Material 10.** Table presenting an overview of the different conclusions reported in the included studies. N = number of studies. **Additional Material 11.** Reference list of included studies.

**Additional file 2: Appendix 1.** Search June 2015.

**Additional file 3: Appendix 2**. Search May 2021.

**Additional file 4:** The filled-in Preferred Reporting Items for Systematic reviews and Meta-Analyses extension for Scoping Reviews (PRISMA-ScR) Checklist.

## Data Availability

The datasets used and/or analysed during the current study are available from the corresponding author on reasonable request.
