# Supplementary material for: Meta-research evaluating redundancy and use of systematic reviews when planning new studies in health research: a scoping review
Source: Syst Rev. 2022 Nov 15;11:241. doi: 10.1186/s13643-022-02096-y (PMC9667610; doi:10.1186/s13643-022-02096-y)
Supplement: Supplementary file 1 — Additional file 1: Additional Material 1. Figure showing number of studies published per year. Additional Material 2. Figure indicates the number of studies from each country, measured as the country affiliation of 1st authors. Additional Material 3. Metrics used in studies evaluating redundancy. N = Number of studies. The total number is higher than the actual number of studies evaluating redundancy, because most studies have used more than one metric. Additional Material 4. Table presenting the metrics used in studies evaluating the use of the EBR approach to minimise or avoid redundancy. N = Number of studies. The total number is higher than the actual number of studies evaluating the use of the EBR approach because most studies have used more than one metric. Additional Material 5. Bibliographic map, the M. Clarke group. Additional Material 6. Bibliographic map, the T.C. Chalmers group. Additional Material 7. Table listing the data materials used in the included studies. (Note that “Primary studies” include papers using various kinds of studies as data material, including some systematic reviews.) Fields marked in light green indicate several studies (6 or more), those in light red indicate few studies (5 or less), and those marked in red indicate no studies. The sum of studies evaluating redundancy/use of EBR approach is higher than the total number, because several studies have evaluated more than one research question. Also, one included paper evaluating the use of the EBR approach [8] used both primary studies and researchers as data material and was therefore counted twice in the table. Additional Material 8. Table listing study designs used in the included studies. Fields marked in light green indicate several studies (6 or more), those in light red indicate few studies (5 or less), and those marked in red indicate no studies. The sum of studies evaluating redundancy/use of EBR is higher than the total number, because several studies have evaluated more th [file 13643_2022_2096_MOESM1_ESM.docx]

**Additional Material**

For “Meta-research evaluating redundancy and use of systematic reviews when planning new studies in health research: a scoping review”

**Additional Material 1**

Figure showing the number of studies published per year.

**Additional Material 2**

This figure indicates the number of studies from each country, measured as the country affiliation of 1^st^ authors.

**Additional Material 3**

Metrics used in the studies evaluating redundancy.

| Method | Metric | N |
| --- | --- | --- |
| Content analysis | Number of MAs that are overlapping | 4 |
|  | Change in trial designs after published research agenda | 2 |
|  | Description of Cum MA finding research waste | 1 |
| Cut-off analysis | Cum MA^§^ - P-value | 16 |
|  | Cum MA - Visual inspection | 4 |
|  | Cum MA – TSA^*^ | 4 |
|  | Cum MA - Extended funnel plot | 1 |
|  | Cum MA - Failsafe ratio | 1 |
|  | Number of studies stopped early for benefit that are followed by new trials | 1 |
|  | Number of trials published after established "high" certainty of evidence | 1 |
|  | Number of trials published after established guidelines | 1 |

N = Number of studies. The total number is higher than the actual number of studies evaluating redundancy because most studies have used more than one metric.

§ Cum MA: Cumulative meta-analysis

* TSA: Trial Sequential Analysis

$ Seq MA: Sequential meta-analysis

**Additional Material 4**

This table presents the metrics used in the studies evaluating the use of an Evidence-Based Research approach to minimise or avoid redundancy.

| Method | Metric | N |
| --- | --- | --- |
| Citation analysis | Number of studies citing SRs | 11 |
|  | Number of studies citing available/relevant SRs | 10 |
|  | Number of similar original studies cited | 8 |
|  | Number of studies citing similar studies | 2 |
|  | Number of studies citing prior SR | 2 |
|  | Number of studies citing SRs or original studies | 2 |
|  | Number of available/relevant SRs that were cited | 1 |
|  | Number and type of studies cited | 1 |
| Content analysis | Number of articles using SRs to justify new study | 12 |
|  | Number of articles using SRs to design new study | 4 |
|  | Number of articles using SRs to place new results in context | 8 |
|  | Number of articles using SRs or original studies to justify, design or interpret new study | 1 |
|  | Description of rationale for conducting new trials | 5 |
|  | Whether SRs were cited verbatim or inferred | 5 |
|  | Number of articles using SRs in Introduction | 3 |
|  | Number of studies cited to justify new study | 1 |
|  | Description of rationale for not citing SRs | 1 |
|  | Prevalence of end user involvement | 1 |
| Survey | Self-reported use of SR | 3 |

N = Number of studies. The total number is higher than the actual number of studies evaluating the use of an Evidence-Based Research approach because most studies have used more than one metric.

**Additional Material 5**


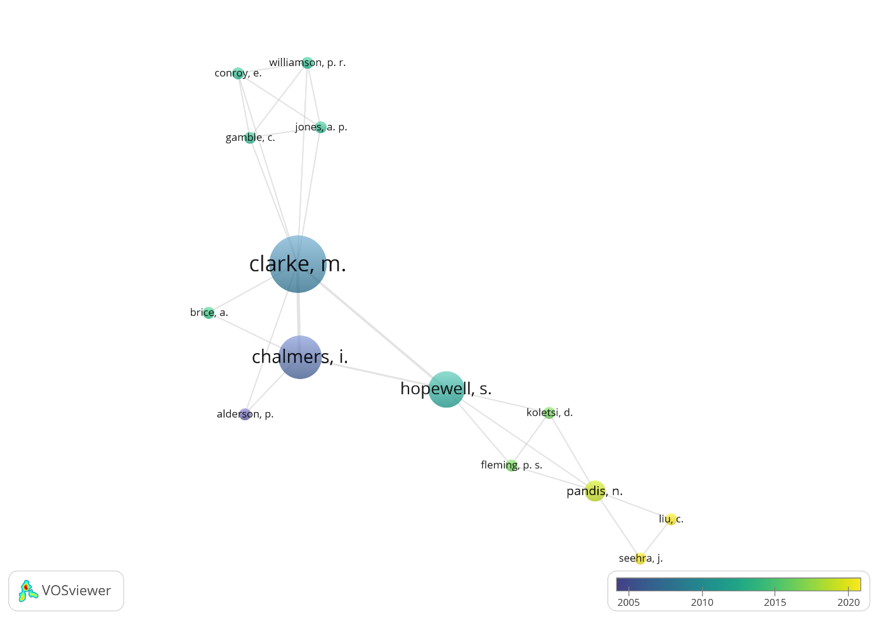


Figure - M Clarke group.

**Additional Material 6**


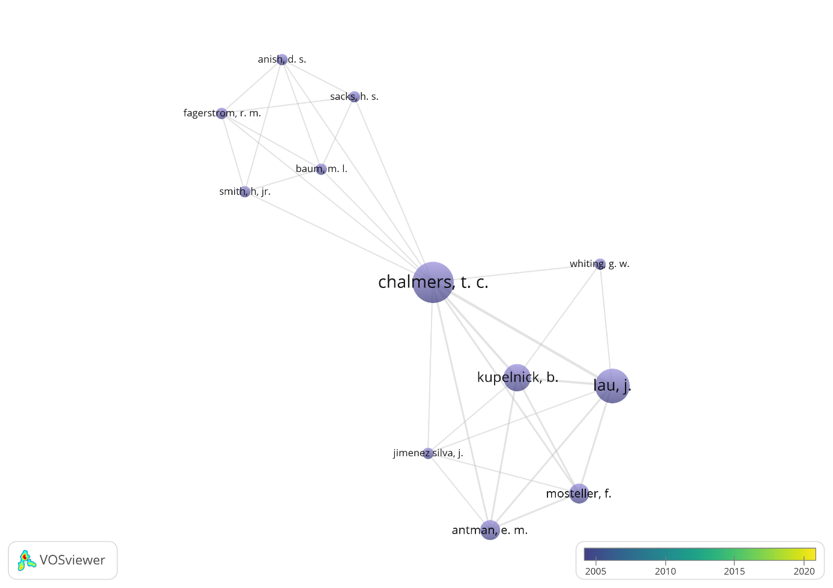


Figure - TC Chalmers-group.

**Additional Material 7**

This table presents the data materials used in the included studies. (Note that “Primary studies” include papers using various kinds of studies as data material, including some systematic reviews.)

|  | Total | Redundancy | Use of the EBR approach | | | | | |
| --- | --- | --- | --- | --- | --- | --- | --- | --- |
|  |  |  | JUSTIFICATION | | DESIGN | | CONTEXT | |
|  |  |  | Earlier studies just. | End user perspec. just. | Earlier studies just. | End user perspec. just. | Earlier studies just. | End user perspec. just. |
| Primary studies | 55 | 28 | 25 | 1 | 9 | 1 | 8 | 1 |
| Systematic reviews | 7 | 6 | 3 | 0 | 0 | 0 | 1 | 0 |
| Funding proposals | 2 | 0 | 2 | 0 | 2 | 0 | 0 | 0 |
| REC* proposals | 2 | 0 | 2 | 0 | 1 | 0 | 0 | 0 |
| Published protocols | 1 | 0 | 1 | 0 | 1 | 0 | 0 | 0 |
| Researchers | 3 | 0 | 2 | 0 | 2 | 0 | 1 | 0 |

Fields marked in light green indicate several studies (6 or more), those in light red indicate few studies (5 or less), and fields marked in red indicate no studies. The sum of studies evaluating redundancy/use of an Evidence-Based Research (EBR) approach is higher than the total number, because several studies have evaluated more than one research question. Also, one included paper evaluating the use of an EBR approach {Ban, 2017 #11364} had used both primary studies and researchers as data material and was therefore counted twice in the table.

*REC: Research Ethic Committee

**Additional Material 8**

This table presents the study designs used in the included studies.

|  | Total | Redundancy | Use of EBR approach | | | | | |
| --- | --- | --- | --- | --- | --- | --- | --- | --- |
|  |  |  | JUSTIFICATION | | DESIGN | | CONTEXT | |
|  |  |  | Earlier studies just. | End user perspec. just. | Earlier studies just. | End user perspec. just. | Earlier studies just. | End user perspec. just. |
| Systematic review | 29 | 27 | 5 | 0 | 3 | 0 | 0 | 0 |
| Cross-sectional study | 38 | 7 | 28 | 1 | 11 | 1 | 10 | 1 |
| Other observational study | 3 | 0 | 2 | 0 | 2 | 0 | 1 | 0 |
| Qualitative study | 0 | 0 | 0 | 0 | 0 | 0 | 0 | 0 |
| Randomised study | 0 | n/a | 0 | 0 | 0 | 0 | 0 | 0 |

Fields marked in light green indicate several studies (6 or more), those in light red indicate few studies (5 or less), and fields marked in red indicate no studies. The sum of studies evaluating redundancy/use of an Evidence-Based Research (EBR) approach is higher than the total number because several studies have evaluated more than one research question. Also, one included paper evaluating the use of an EBR approach {Ban, 2017 #11364} had used both primary studies and researchers as data material and was therefore counted twice in the table.

**Additional Material 9**

This table presents the analysis methods used in the included studies.

|  | Total | Redundancy | Use of EBR approach | | | | | |
| --- | --- | --- | --- | --- | --- | --- | --- | --- |
|  |  |  | JUSTIFICATION | | DESIGN | | CONTEXT | |
|  |  |  | Earlier studies just. | End user perspec. just. | Earlier studies just. | End user perspec. just. | Earlier studies just. | End user perspec. just. |
| Citation analysis | 37 | 0 | 31 | 0 | 13 | 0 | 9 | 0 |
| Content analysis | 34 | 7 | 22 | 1 | 10 | 1 | 10 | 1 |
| Cut-off analysis | 27 | 27 | n/a | | | | | |
| Survey | 3 | 0 | 2 | 0 | 2 | 0 | 1 | 0 |

Fields marked in light green indicate several studies (6 or more), those in light red indicate few studies (5 or less), and fields marked in red indicate no studies. Note that many of the included studies have used more than one analysis method and investigated more than one research question. For that reason, studies have been counted several times in the table, and the sum of studies is much higher than the total number of included papers.

**Additional Material 10**

This table presents an overview of the different conclusions reported in the included studies.

| Redundancy | | Use of EBR approach | | | | | |
| --- | --- | --- | --- | --- | --- | --- | --- |
|  |  | JUSTIFICATION | | DESIGN | | CONTEXT | |
| Conclusion | N | Concl. | N | Concl. | N | Concl. |  |
| Identified redundancy among similar clinical studies | 23 | No or poor citation of original similar studies | 6 | No or poor use of SRs to inform design | 6 | No or poor use of SR to place new results in context | 7 |
| Identified redundancy among similar SRs | 3 | No or poor use of SRs to inform justification of new study | 15 | Systematic Reviews can help design new studies | 3 | The use of SRs to inform analysis of clinical trials is widely considered desirable | 1 |
| Cumulative meta-analyses can help reduce redundancy | 3 | Large variation in how a RCT is justified | 1 | The use of SRs to inform design and conduct of clinical trials is widely considered desirable | 1 |  |  |
| No or poor citation of original similar studies | 2 | SRs can help justifying new studies | 1 |  |  |  |  |
| Poor quality of studies leads to redundancy | 1 | Cumulative meta-analyses can help reduce redundancy | 3 |  |  |  |  |
| SRs showing effect stimulates increase in trial production and publication | 1 | Uncertainty of the question was not or poorly documented | 1 |  |  |  |  |
| Identified continuous use of harmful treatment based upon a cumulative MA | 1 | No or poor use of end users in clinical studies | 1 |  |  |  |  |
| Trials stopped early for benefit were followed by subsequent trials addressing a similar question | 1 | Better use of SRs to inform justification of new study | 1 |  |  |  |  |
|  |  | Citation network of related trials are often disconnected | 1 |  |  |  |  |

N = number of studies.

**Additional Material 11**

Reference list of included studies

1. Antman EM, Lau J, Kupelnick B, Mosteller F, Chalmers TC. A comparison of results of meta-analyses of randomized control trials and recommendations of clinical experts. Treatments for myocardial infarction. JAMA. 1992;268(2):240-8.
2. Ban JW, Wallace E, Stevens R, Perera R. Why do authors derive new cardiovascular clinical prediction rules in the presence of existing rules? A mixed methods study. PLoS One. 2017;12(6):e0179102.
3. Baum ML, Anish DS, Chalmers TC, Sacks HS, Smith H, Jr., Fagerstrom RM. A survey of clinical trials of antibiotic prophylaxis in colon surgery: evidence against further use of no-treatment controls. N Engl J Med. 1981;305(14):795-9.
4. Bauman A, Milton K, Kariuki M, Fedel K, Lewicka M. Is there sufficient evidence regarding signage-based stair use interventions? A sequential meta-analysis. BMJ Open. 2017;7(11):e012459.
5. Bhurke S, Cook A, Tallant A, Young A, Williams E, Raftery J. Using systematic reviews to inform NIHR HTA trial planning and design: a retrospective cohort. BMC Med Res Methodol. 2015;15:108.
6. Blanco-Silvente L, Castells X, Garre-Olmo J, Vilalta-Franch J, Saez M, Barcelo MA, et al. Study of the strength of the evidence and the redundancy of the research on pharmacological treatment for Alzheimer's disease: a cumulative meta-analysis and trial sequential analysis. Eur J Clin Pharmacol. 2019;75(12):1659-67.
7. Bolland MJ, Avenell A, Grey A. Assessment of research waste part 1: an exemplar from examining study design, surrogate and clinical endpoints in studies of calcium intake and vitamin D supplementation. BMC Med Res Methodol. 2018;18(1):103.
8. Bolland MJ, Grey A, Avenell A. Assessment of research waste part 2: wrong study populations- an exemplar of baseline vitamin D status of participants in trials of vitamin D supplementation. BMC Med Res Methodol. 2018;18(1):101.
9. Brockington I. Citation analysis of puerperal and menstrual psychosis. Arch Womens Ment Health. 2017;20(1):49-53.
10. Chalmers TC, Lau J. Changes in clinical trials mandated by the advent of meta-analysis. Stat Med. 1996;15(12):1263-8; discussion 9-72.
11. Chapman SJ, Aldaffaa M, Downey CL, Jayne DG. Research waste in surgical randomized controlled trials. Br J Surg. 2019;106(11):1464-71.
12. Chiu L, Chow R, DeAngelis C, Lock M, Simone CB, 2nd. Secondary and cumulative meta-analysis of olanzapine for antiemetic prophylaxis for chemotherapy-induced nausea and vomiting: do we still need to study its effectiveness? Ann Palliat Med. 2021;10(3):2540-7.
13. Chow JT, Lam K, Naeem A, Akanda ZZ, Si FF, Hodge W. The pathway to RCTs: how many roads are there? Examining the homogeneity of RCT justification. Trials. 2017;18(1):51.
14. Chow R, Aapro M, Navari RM, Gralla R, Chiu N, Chiu L, et al. Do we still need to study palonosetron for chemotherapy-induced nausea and vomiting? A cumulative meta-analysis. Crit Rev Oncol Hematol. 2019;142:164-86.
15. Chow R, Bruera E, Arends J, Walsh D, Strasser F, Isenring E, et al. Enteral and parenteral nutrition in cancer patients, a comparison of complication rates: an updated systematic review and (cumulative) meta-analysis. Support Care Cancer. 2020;28(3):979-1010.
16. Clarke M, Alderson P, Chalmers I. Discussion sections in reports of controlled trials published in general medical journals. JAMA. 2002;287(21):2799-801.
17. Clarke M, Brice A, Chalmers I. Accumulating Research: A Systematic Account of How Cumulative Meta-Analyses Would Have Provided Knowledge, Improved Health, Reduced Harm and Saved Resources. PLoS One. 2014;9(7).
18. Clarke M, Chalmers I. Discussion sections in reports of controlled trials published in general medical journals: islands in search of continents? JAMA. 1998;280(3):280-2.
19. Clarke M, Hopewell S. Many reports of randomised trials still don't begin or end with a systematic review of the relevant evidence - BESTILT. J Bahrain Med Soc. 2013;24:145-48.
20. Clarke M, Hopewell S, Chalmers I. Reports of clinical trials should begin and end with up-to-date systematic reviews of other relevant evidence: a status report. J R Soc Med. 2007;100(4):187-90.
21. Clarke M, Hopewell S, Chalmers I. Clinical trials should begin and end with systematic reviews of relevant evidence: 12 years and waiting. Lancet. 2010;376(9734):20-1.
22. Clayton GL, Smith IL, Higgins JPT, Mihaylova B, Thorpe B, Cicero R, et al. The INVEST project: investigating the use of evidence synthesis in the design and analysis of clinical trials. Trials. 2017;18(1):219.
23. Conde-Taboada A, Aranegui B, Garcia-Doval I, Davila-Seijo P, Gonzalez-Castro U. The use of systematic reviews in clinical trials and narrative reviews in dermatology: is the best evidence being used? Actas Dermosifiliogr. 2014;105(3):295-9.
24. Coomarasamy A, Thangaratinam S, Gee H, Khan KS. Progesterone for the prevention of preterm birth: a critical evaluation of evidence. Eur J Obstet Gynecol Reprod Biol. 2006;129(2):111-8.
25. Cooper NJ, Jones DR, Sutton AJ. The use of systematic reviews when designing studies. Clin Trials. 2005;2(3):260-4.
26. De Meulemeester J, Fedyk M, Jurkovic L, Reaume M, Dowlatshahi D, Stotts G, et al. Many randomized clinical trials may not be justified: a cross-sectional analysis of the ethics and science of randomized clinical trials. J Clin Epidemiol. 2018;97:20-5.
27. Engelking A, Cavar M, Puljak L. The use of systematic reviews to justify anaesthesiology trials: A meta-epidemiological study. European Journal of Pain. 2018;22(10):1844-9.
28. Fergusson D, Glass KC, Hutton B, Shapiro S. Randomized controlled trials of aprotinin in cardiac surgery: could clinical equipoise have stopped the bleeding? Clin Trials. 2005;2(3):218-29; discussion 29-32.
29. Fergusson D, Monfaredi Z, Pussegoda K, Garritty C, Lyddiatt A, Shea B, et al. The prevalence of patient engagement in published trials: a systematic review. Research Involvement and Engagement. 2018;4(1):17.
30. Goudie AC, Sutton AJ, Jones DR, Donald A. Empirical assessment suggests that existing evidence could be used more fully in designing randomized controlled trials. J Clin Epidemiol. 2010;63(9):983-91.
31. Habre C, Tramer MR, Popping DM, Elia N. Ability of a meta-analysis to prevent redundant research: Systematic review of studies on pain from propofol injection. BMJ (Online). 2014;349(1305).
32. Helfer B, Prosser A, Samara MT, Geddes JR, Cipriani A, Davis JM, et al. Recent meta-analyses neglect previous systematic reviews and meta-analyses about the same topic: a systematic examination. BMC Med. 2015;13:82.
33. Henderson WG, Moritz T, Goldman S, Copeland J, Sethi G. Use of cumulative meta-analysis in the design, monitoring, and final analysis of a clinical trial: a case study. Control Clin Trials. 1995;16(5):331-41.
34. Hoderlein X, Moseley AM, Elkins MR. Citation of prior research has increased in introduction and discussion sections with time: A survey of clinical trials in physiotherapy. Clin Trials. 2017;14(4):372-80.
35. Ivers NM, Grimshaw JM, Jamtvedt G, Flottorp S, O'Brien MA, French SD, et al. Growing literature, stagnant science? Systematic review, meta-regression and cumulative analysis of audit and feedback interventions in health care. J Gen Intern Med. 2014;29(11):1534-41.
36. Jia Y, Wen J, Qureshi R, Ehrhardt S, Celentano DD, Wei X, et al. Effect of redundant clinical trials from mainland China evaluating statins in patients with coronary artery disease: cross sectional study. BMJ. 2021;372:n48.
37. Johnson AL, Walters C, Gray H, Torgerson T, Checketts JX, Boose M, et al. The use of systematic reviews to justify orthopaedic trauma randomized controlled trials: A cross-sectional analysis. Injury. 2020;51(2):212-7.
38. Jones AP, Conroy E, Williamson PR, Clarke M, Gamble C. The use of systematic reviews in the planning, design and conduct of randomised trials: a retrospective cohort of NIHR HTA funded trials. BMC Med Res Methodol. 2013;13:50.
39. Joseph PD, Caldwell PH, Barnes EH, Hynes K, Choong CS, Turner S, et al. Completeness of protocols for clinical trials in children submitted to ethics committees. J Paediatr Child Health. 2019;55(3):291-8.
40. Juni P, Nartey L, Reichenbach S, Sterchi R, Dieppe PA, Egger M. Risk of cardiovascular events and rofecoxib: cumulative meta-analysis. Lancet [Internet]. 2004; 364(9450):[2021-9 pp.].
41. Ker K, Edwards P, Perel P, Shakur H, Roberts I. Effect of tranexamic acid on surgical bleeding: systematic review and cumulative meta-analysis. BMJ. 2012;344:e3054.
42. Ker K, Roberts I. Exploring redundant research into the effect of tranexamic acid on surgical bleeding: further analysis of a systematic review of randomised controlled trials. BMJ Open. 2015;5(8):e009460.
43. Lau J, Antman EM, Jimenez-Silva J, Kupelnick B, Mosteller F, Chalmers TC. Cumulative meta-analysis of therapeutic trials for myocardial infarction. N Engl J Med. 1992;327(4):248-54.
44. Love R, Adams J, van Sluijs EMF, Foster C, Humphreys D. A cumulative meta-analysis of the effects of individual physical activity interventions targeting healthy adults. Obes Rev. 2018;19(8):1164-72.
45. Murad MH, Guyatt GH, Domecq JP, Vernooij RWM, Erwin PJ, Meerpohl JJ, et al. Randomized trials addressing a similar question are commonly published after a trial stopped early for benefit. J Clin Epidemiol. 2017;82:12-9.
46. Paludan-Müller AS, Ogden MC, Marquardsen M, Vive J, Jørgensen KJ, Gøtzsche PC. Do protocols for new randomised trials take previous similar trials into account? Cohort study of contemporary trial protocols. BMJ Open. 2019;9(11):e026661.
47. Pandis N, Fleming PS, Koletsi D, Hopewell S. The citation of relevant systematic reviews and randomised trials in published reports of trial protocols. Trials. 2016;17(1):581.
48. Park JH, Eisenhut M, van der Vliet HJ, Shin JI. Statistical controversies in clinical research: overlap and errors in the meta-analyses of microRNA genetic association studies in cancers. Annals of Oncology. 2017;28(6):1169-82.
49. Poolman RW, Farrokhyar F, Bhandari M. Hamstring tendon autograft better than bone patellar-tendon bone autograft in ACL reconstruction: a cumulative meta-analysis and clinically relevant sensitivity analysis applied to a previously published analysis. Acta Orthop. 2007;78(3):350-4.
50. Rauh S, Nigro T, Sims M, Vassar M. The use of systematic reviews to justify randomized controlled trials in obstetrics & gynecology publications. Eur J Obstet Gynecol Reprod Biol. 2020;252:627-8.
51. Riaz IB, Khan MS, Riaz H, Goldberg RJ. Disorganized Systematic Reviews and Meta-analyses: Time to Systematize the Conduct and Publication of These Study Overviews? The American Journal of Medicine. 2016;129(3):339.e11-e18.
52. Robinson KA, Dunn AG, Tsafnat G, Glasziou P. Citation networks of related trials are often disconnected: Implications for bidirectional citation searches. Journal of Clinical Epidemiology. 2014;67(7):793-9.
53. Robinson KA, Goodman SN. A systematic examination of the citation of prior research in reports of randomized, controlled trials. Annals of Internal Medicine. 2011;154(1):50-5.
54. Rosenthal R, Bucher HC, Dwan K. The Use of Systematic Reviews When Designing and Reporting Surgical Trials. Ann Surg. 2017;265(4):e35-e6.
55. Ross JS, Madigan D, Hill KP, Egilman DS, Wang Y, Krumholz HM. Pooled analysis of rofecoxib placebo-controlled clinical trial data: lessons for postmarket pharmaceutical safety surveillance. Arch Intern Med. 2009;169(21):1976-85.
56. Sawin VI, Robinson KA. Biased and inadequate citation of prior research in reports of cardiovascular trials is a continuing source of waste in research. J Clin Epidemiol. 2016;69:174-8.
57. Seehra J, Liu C, Pandis N. Citation of prior systematic reviews in reports of randomized controlled trials published in dental speciality journals. J Dent. 2021;109:103658.
58. Shepard S, Wise A, Johnson BS, Sajjadi NB, Hartwell M, Vassar M. Are randomized controlled trials in urology being conducted with justification? J Osteopath Med. 2021;121(8):665-71.
59. Sheth U, Simunovic N, Tornetta P, 3rd, Einhorn TA, Bhandari M. Poor citation of prior evidence in hip fracture trials. The Journal of bone and joint surgery American volume. 2011;93(22):2079-86.
60. Sigurdson MK, Khoury MJ, Ioannidis JPA. Redundant meta-analyses are common in genetic epidemiology. Journal of Clinical Epidemiology. 2020;127:40-8.
61. Sinclair JC. Meta-analysis of randomized controlled trials of antenatal corticosteroid for the prevention of respiratory distress syndrome: discussion. Am J Obstet Gynecol. 1995;173(1):335-44.
62. Siontis KC, Hernandez-Boussard T, Ioannidis JP. Overlapping meta-analyses on the same topic: survey of published studies. BMJ. 2013;347:f4501.
63. Smith AJ, Goodman NW. The hypertensive response to intubation. Do researchers acknowledge previous work? Can J Anaesth. 1997;44(1):9-13.
64. Storz-Pfennig P. Potentially unnecessary and wasteful clinical trial research detected in cumulative meta-epidemiological and trial sequential analysis. J Clin Epidemiol. 2017;82:61-70.
65. Torgerson T, Evans S, Johnson BS, Vassar M. The use of systematic reviews to justify phase III ophthalmology trials: an analysis. Eye (Lond). 2020;34(11):2041-7.
66. Vergara-Merino L, Verdejo C, Franco JVA, Escobar Liquitay C, Urrútia G, Klabunde R, et al. Registered trials address questions already answered with high-certainty evidence: A sample of current redundant research. Journal of Clinical Epidemiology. 2021;134:89-94.
67. Verhagen AP, Ferreira M, Reijneveld-van de Vendel EAE, Teirlinck CH, Runhaar J, van Middelkoop M, et al. Do we need another trial on exercise in patients with knee osteoarthritis?: No new trials on exercise in knee OA. Osteoarthritis Cartilage. 2019;27(9):1266-9.
68. Walters C, Torgerson T, Fladie I, Clifton A, Meyer C, Vassar M. Are randomized controlled trials being conducted with the right justification? Journal of Evidence-Based Medicine. 2020;13(3):181-2.
69. Whiting GW, Lau J, Kupelnick B, Chalmers TC. Trends in Inflammatory Bowel Disease Therapy: A Meta-Analytic Approach. Canadian Journal of Gastroenterology. 1995;9:283251.
