# Supplementary material for: Meta-research evaluating redundancy and use of systematic reviews when planning new studies in health research: a scoping review
Source: Syst Rev. 2022 Nov 15;11:241. doi: 10.1186/s13643-022-02096-y (PMC9667610; doi:10.1186/s13643-022-02096-y)
Supplement: Supplementary file 3 — Additional file 3: Appendix 2. Search May 2021. [file 13643_2022_2096_MOESM3_ESM.docx]

Appendix 2

# SEARCH CONDUCTED ON 26 MAY 2021

**Source**: MEDLINE (OVID)

**Time span:** all time (final search limited to 2019 – current)

**Conducted by:** Ane Gjerland, 26^th^ of May 2021

# Part 1: EBR

This part includes all basic terms relating to Evidence-Based Research and meta-research studies. As any study using these terms may be relevant, all have been included as they are regardless of the low precision.

| **Search #** | **Search terms** | **Number of hits (2021)** |
| --- | --- | --- |
|  | evidence-based research.ti,ab. | 943 |
|  | research on research.ti,ab. | 85 |
|  | meta-research.ti,ab. | 82 |
|  | metaresearch.ti,ab. | 8 |
|  | cumulative meta-analys*.ti,ab. | 624 |
|  | cumulative metaanalys*.ti,ab. | 6 |
|  | ***1 or 2 or 3 or 4 or 5 or 6*** | ***1741*** |

# Part 2: Redundancy

This part focuses on the topic of redundant research, aiming to identify studies mentioning terms such as “research waste” and “redundant trials”.

The approach taken is to combine groups of terms that are typically used in titles and abstracts of studies on redundancy. In order to simplify the search, groups of terms were combined instead of combining each possible combination of each term. The number of hits for each of the large groups of terms (searches #6, #7, and #8) is therefore not important; what is important is the number of hits for the combinations of these terms marked with a yellow background.

| **Search #** | **Search name / explanation** | **Search terms** | **Number of hits (2021)** |
| --- | --- | --- | --- |
|  | REDUNDANCY | (redundan* or futil* or (waste* not (wastewater or wastes or waste-to-energy or waste-derived or "waste disposal")) or unnecessary or overlap* or inefficien* or (lack* adj2 novelt*) or ("no new" adj3 knowl*) or "evaluat* identical associations" or "potential duplication" or (("summary effect" or "lack of" or "did not" or "has not") adj3 change*) or ("limited value" adj2 "new") or “value of information analysis”).ti,ab. | 513921 |
|  | STUDY | (study or studies or trial or trials or RCT* or "randomi#ed controlled trial*" or project or projects or report or reports or protocol* or evidence or research or publication* or published).ti,ab. | 13928838 |
|  | REVIEW | ("systematic review*" or meta-analys* or metaanalys* or meta-synthes* or metasynthes* or "evidence synthes*" or "systematic literature search" or "trial sequential analysis" or (chronological adj2 review)).ti,ab. | 316555 |
|  | *(REDUNDANCY ADJ1 STUDY)* | *8 adj1 9* | 1777 |
|  | *(REDUNDANCY ADJ3 REVIEW)* | *8 adj3 10* | 246 |
|  | (STUDY ADJ5 REVIEW) | 9 adj5 10 | 140629 |
|  | *(REVIEW ADJ5 STUDY) ADJ5 REDUNDANCY* | *13 adj5 8* | *157* |
|  | ***(REDUNDANCY ADJ1 STUDY) OR (REDUNDANCY ADJ3 REVIEW) OR (REVIEW ADJ5 STUDY) ADJ5 REDUNDANCY)*** | ***11 or 12 or 14*** | ***2059*** |

# Part 3: Justify

The same approach as for part 2 was initially applied in order to identify studies on justification/design/context. However, here the approach unfortunately did not work, resulting in too much noise.

So instead, the focus here shifted to using the specific terms used by already included studies to create search strings. The resulting search strings are therefore very specific, resulting in a low number of hits but a high ratio of relevant studies.

| **Search #** | **Search terms** | **Number of hits (2021)** |
| --- | --- | --- |
|  | ("sample size" adj2 citation).ti,ab. | 3 |
|  | ("prior research" adj2 citation).ti,ab. | 3 |
|  | ((avoidable or preventable) adj2 "design limitation*").ti,ab. | 3 |
|  | (question* adj3 "already answered").ti,ab. | 12 |
|  | ((sufficient or satisfactory or acceptable) adj2 "evidence base").ti,ab. | 58 |
|  | ((justifying or justified) adj (study or studies or trial or trials or RCT* or "randomi#ed controlled trial*" or project or projects or report or reports or protocol* or evidence or research or publication* or published)).ti,ab. | 74 |
|  | (("rationale for conducting" or "justification for conducting" or "justif* conducting") adj2 (study or studies or trial or trials or RCT* or "randomi#ed controlled trial*" or project or projects or report or reports or protocol* or evidence or research or publication* or published)).ti,ab. | 64 |
|  | ("inadequate* plan*" adj5 (study or studies or trial or trials or RCT* or "randomi#ed controlled trial*" or project or projects or report or reports or protocol* or evidence or research or publication* or published)).ti,ab. | 8 |
|  | (("use of systematic review*" or "systematic review us*" or "cited systematic review" or "cited a systematic review") adj3 (justif* or rationale or reason or inform or lack* or absence)).ti,ab. | 13 |
|  | ((consider* or "into account") adj1 ((prior or previous or existing or old or older or earlier or established or existing or related or similar) adj (trial or trials or RCT* or "randomi#ed controlled trial*" or protocol* or evidence or publication* or published))).ti,ab. | 52 |
|  | **16 or 17 or 18 or 19 or 20 or 21 or 22 or 23 or 24 or 25** | **282** |

# Part 4: Total

The combination of parts 1-3, limited to recent studies only.

| **Search #** | **Search terms** | **Number of hits (2021)** |
| --- | --- | --- |
|  | **7 or 15 or 26** | **4055** |
|  | limit 27 to yr="2019 -Current" | *1005* |
|  | After EndNote de-duplication | *998* |
|  | After Rayyan de-duplication | *996* |
